# Fecal Impaction With Multisystem Organ Involvement

**DOI:** 10.5811/cpcem.2016.12.32754

**Published:** 2017-01-24

**Authors:** Joseph Pepe, Melissa M. Murphy, Kevin P. O’Connell, Christopher P. Zabbo

**Affiliations:** * Kent Hospital, Department of Emergency Medicine, Warwick, Rhode Island; † Kent Hospital, Department of Surgery, Warwick, Rhode Island

## Abstract

Fecal impactions are a common complaint in the emergency department (ED) population. The potential for significant derangement in physiologic processes of other organ systems is often underappreciated. A 19-year-old male, previously healthy, presented to the ED at our institution with complaint of abdominal pain, which was found to be secondary to severe fecal impaction. In the search for alternative diagnoses, imaging was performed, which revealed effects on multiple other organ systems. This case illustrates the secondary effects of a severe fecal impaction. The emergency physician must be aware of these consequences, as the opportunity to review labs and imaging is not often provided in the standard workup of these patients.

## INTRODUCTION

Fecal impaction is a common complaint addressed in the emergency department (ED). Serious complications are not often observed, and the problem is commonly resolved quickly with a manual disimpaction. Most patients do not require laboratory evaluation or advanced imaging.^1^ Occasionally the search for alternative diagnoses requires diagnostic testing beyond the history and physical exam. Imaging may demonstrate secondary diagnoses related to the physiologic disruption caused by severe retention of fecal material.

## CASE REPORT

A 19-year-old male presented to our institution complaining of abdominal pain for two weeks. He reported that he initially felt constipated and started taking over-the-counter medications to self-treat his condition. These medications included polyethylene glycol, various stool softeners, and a colonoscopy preparation product he received from a family member. One week prior to presentation, he developed watery stools and discontinued all medications. Since that time he reported continued watery, non-bloody stools of small volume approximately twice daily. The patient noted a remote history of constipation as a child, requiring manual disimpaction. Since that time, he had been taking polyethylene glycol daily without further issues. There was no personal or family history of Hirschsprung’s disease. He denied any nausea, vomiting, or fevers. On further history taking he did admit to using his family member’s oxycodone over the prior several weeks. There was no recent antibiotic use and travel history was unremarkable. Physical examination was significant for a distended and firm abdomen, hypoactive bowel sounds, and mild generalized tenderness to palpation without peritoneal signs. Rectal exam revealed a small amount of brown watery stool externally and hard intraluminal stool in the proximal rectum felt with the fingertip. Fecal occult blood testing was negative. The patient complained of severe rectal pain, limiting further evaluation of the rectal vault more proximally.

Laboratory values were notable for creatinine 1.03 mg/dL, total bilirubin 1.1 mg/dL, and direct bilirubin 0.29 mg/dL. The remainder of the complete metabolic panel was within normal limits, as was a complete blood count and urinalysis. Due to the hard, distended abdomen on exam, there was concern for the possibility of serious intra-abdominal pathology.

**Image 1:**
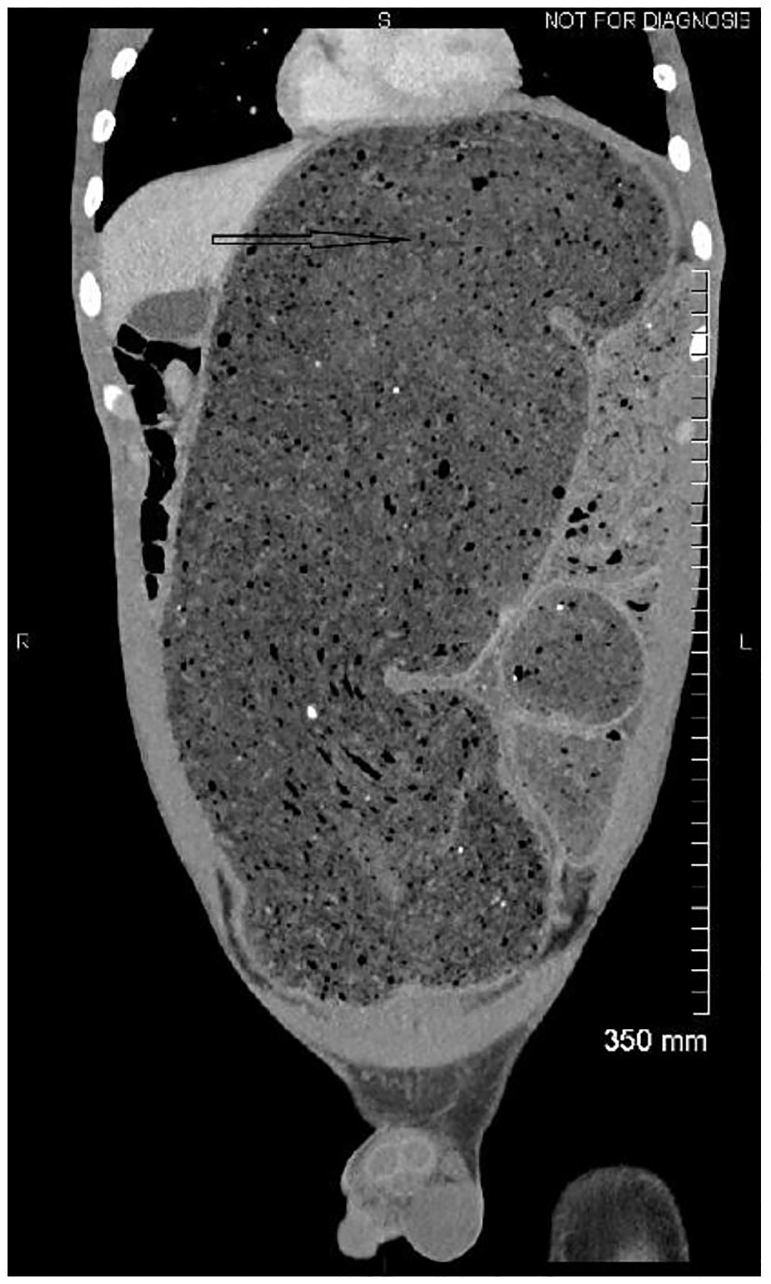
Coronal section of the abdomen by computed tomography displaying extent of the extensive stool burden.

**Image 2:**
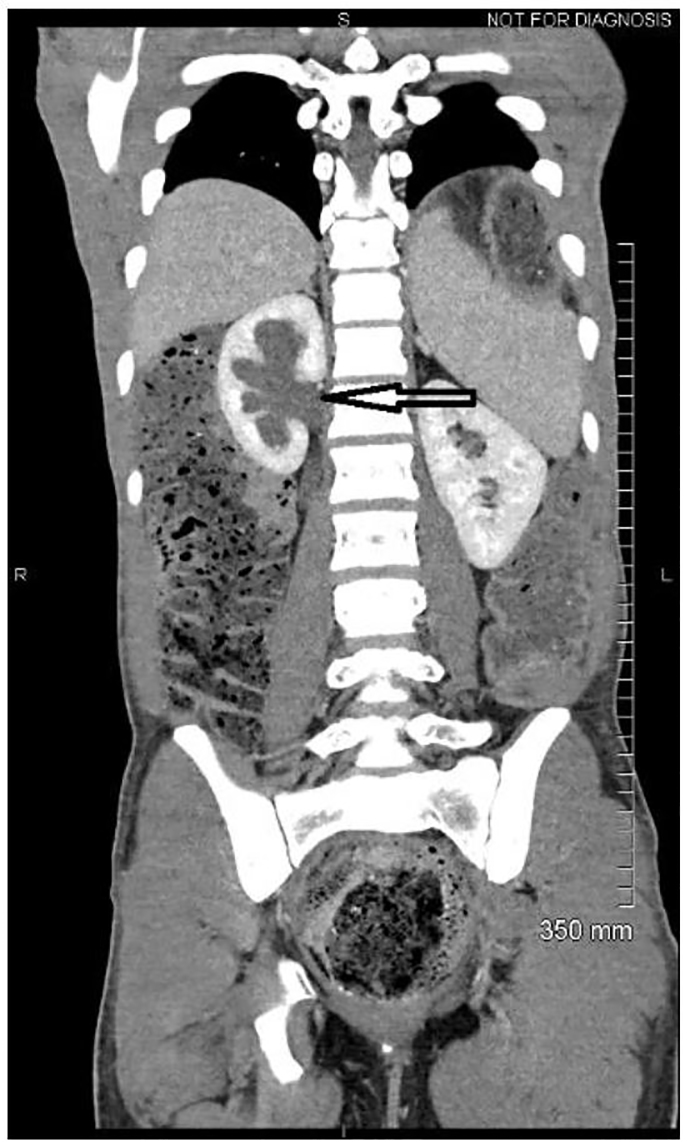
Coronal section showing right hydronephrosis (arrow) due to obstructive uropathy from fecal material.

**Image 3:**
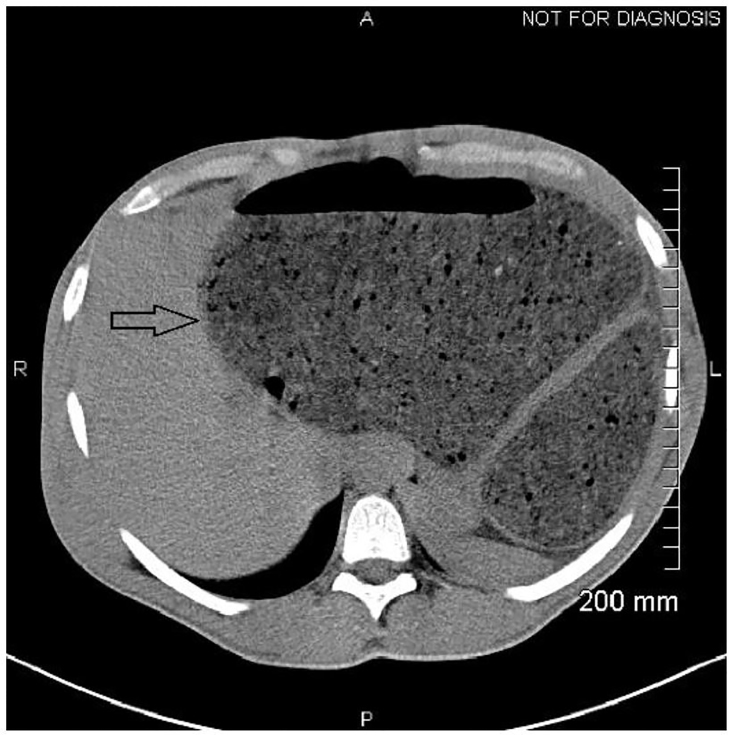
Distended colon exerting mass effect on the liver (arrow).

It was felt that computed tomography of the abdomen and pelvis would provide the necessary information to rule out alternative diagnoses, as well as further delineate the location of a possible bowel obstruction. Imaging revealed extensive retained fecal material, which distended the sigmoid colon up to 18.2 centimeters and extended into the upper abdomen, exerting mass effect on multiple solid organs (Image 1). Bilateral hydronephrosis was observed secondary to mass compression of bilateral ureters (Image 2). The bladder was displaced anteriorly and superiorly from its expected position. In addition, the colon can be seen compressing the left hepatic lobe (Image 3).

Colorectal surgery was consulted and initially attempted manual disimpaction in the ED. However, this was aborted secondary to severe patient discomfort. Due to the extensive nature of the impaction, treatment with naloxone was not considered a viable option. The risk of bowel perforation discouraged further attempts at relieving the impaction under conscious sedation at the bedside. Thus, the patient was taken to the operating room for manual disimpaction under general anesthesia. The large stool ball in the proximal rectum needed to be broken into pieces and removed manually. Greater than 15 pounds of soft stool were evacuated. The patient was admitted for observation and was discharged the following hospital day with instructions to follow up.

## DISCUSSION

Constipation is a common complaint in ED patients. The prevalence of constipation in the community is estimated to be around 16%.^1^ Constipation may be attributed to stool quality, colonic motility, or outlet obstruction. As symptoms progress and become more severe, patients may develop obstipation. This can further develop into a large bowel obstruction, which can be complicated by perforation if not recognized and treated expeditiously.

Colonic contractions normally propel stool several times a day, often postprandially. It is known that voluntary suppression of defecation is a risk factor for constipation due to decreasing bowel motility and a blunted motor response to eating.^2^ Additionally, a number of other etiologies and contributing factors to constipation exist, including low fiber intake, dehydration, spinal cord injury, stroke, dementia, advanced age, and Hirschsprung’s disease. Constipation is a common medication side effect and is associated with opiate analgesics, anticholinergics, antacids, iron, and calcium-channel blockers. Often, fecal impaction is a consequence of chronic or untreated constipation.

Evaluation of the patient with possible fecal impaction should start with a detailed history, including special attention to the risk factors described above. A key element in the patient interview is eliciting a history of previous impaction, which is found in up to 39% of patients. 3 Physical exam findings may include signs of dehydration, diffuse abdominal tenderness, and fullness in the left lower quadrant corresponding to fecal matter collecting in the rectosigmoid colon.

A wide array of complications attributed to fecal impaction has been noted in the literature, primarily published as case reports. A recent systematic review detailed the relative proportions of these complications.^4^ The most common complications observed were secondary to local compression of the colon and included intestinal perforation, obstruction, and ulceration. Interestingly, a significant number of cases involved complications without direct gastrointestinal damage. The highest number of cases involved obstructive uropathy, which was observed in our patient. Additional cases noted urinary bladder damage, as well as compression of nerve and vascular structures. Although our patient was found to have hepatic compression with mild hyperbilirubinemia and ascites, the effect on other organ systems has been documented in the literature. Consideration of these uncommon but serious medical conditions should be a part of clinical decision-making in these patients. The recognition of the secondary effects is important, in that these effects must be followed to resolution to exclude underlying pathology in those organ systems that may be obscured by the fecal impaction.

Emergency physicians are trained to consider the most serious diagnoses in their differential decision-making first. Constipation with fecal impaction is often thought to be a benign condition; however, our case demonstrates that fecal impaction can have effects on multiple organ systems. Despite our patient’s young age, there was observable impact on both his renal and hepatic systems. The patient recovered quickly without any known lasting morbidity; however, the consequences may be more significant in patients of advanced age or with other comorbid conditions. It is important for the emergency physician to be aware of these findings and consider additional work-up in patients with high risk of complications. Additionally, ensuring appropriate follow-up with a primary care physician and instituting an appropriate bowel regimen can prevent further instances and subsequent complications.
